# Association between condom use and perspectives on contraceptive responsibility in different sexual relationships among sexually active college students in China: A cross-sectional study

**DOI:** 10.1097/MD.0000000000013879

**Published:** 2019-01-04

**Authors:** Lu Long, Yutong Han, Longxia Tong, Zhenhua Chen

**Affiliations:** a Department of Epidemiology and Biostatistics, West China School of Public Health; b Key Laboratory of Birth Defects and Related Diseases of Women and Children, Sichuan University; c Chengdu Municipal Center for Disease Control and Prevention, Chengdu, Sichuan, China.

**Keywords:** college students, contraceptive responsibility, sexual relationships

## Abstract

China has increasing incidence of unplanned pregnancies among college students these years. Increasing students’ sense of responsibility to protect sexual partners may help reduce the rate of unplanned pregnancies.

A self-administered questionnaire was distributed to students from 3 colleges in Sichuan, China. Among them, 559 of male students and 267 of female students were included in this study. The questionnaire collected participants’ characteristics, usage of condom, and investigated male and female students’ perceptions of who should be responsible for contraception use.

We found that relationship status was closely related to students perceiving responsibility for reproductive health and condom using. Male students who were in casual relationships during their most recent sexual encounter and shared responsibility for contraception were more likely to use condoms than other male students (*P* < .001). Female students who were in steady relationships during their most recent sexual encounter and shared responsibility for contraception were more likely to use condoms than other female students (*P* = .007). The multivariate analysis revealed condom use was associated with greater odds of sharing responsibility for contraceptive use in different types of sexual relationships.

Improving students’ attitudes toward responsibility for contraception may increase condom use among students at risk for unplanned pregnancies. Programs providing targeted health education and services may help reduce the rate of unplanned pregnancies among students in China.

## 1 Introduction

China has experienced dramatic social changes over the past few decades. Social and economic developments have changed attitudes of young Chinese people toward sexuality, particularly among college students.^[1]^ Previous studies on sexuality and sexual behavior among Chinese college students showed that an increasing number of these students have had sex before marriage; more than one-quarter have never had steady sexual partners.^[2–4]^ In addition, approximately 50% of students used condoms for fertility regulation when having sexual intercourse with their sexual partners.^[3–5]^ Extensive research has been conducted on the role of females and males in reproductive health decision-making, especially among teen populations.^[6–10]^ Several studies have shown that increasing students’ sense of contraceptive responsibility to protect sexual partners may help reduce the high rate of unplanned pregnancies among students.^[11,12]^

Contraceptive responsibility is an understudied area that may help health educators and clinicians design more effective pregnancy and sexually transmitted infection (STI) prevention programs. People who share contraceptive responsibility are more likely to attend medical appointments with partners and remind each other to use condoms on a daily basis.^[13]^ Shared responsibility also helps with the financial costs of contraception.^[14]^ Several recent international studies that focused on the roles of men and women in student pregnancy found the pregnancy resolution was related to students’ views about contraceptive responsibility.^[13,15]^ However, no studies in China have examined the association between college students’ perceptions of contraceptive responsibility and status of condom use among them.

Previous studies showed that students who were in relationships were more likely to use contraceptives than single students, with this attributed to the association between reduced communication in early casual relationships and risky sexual behaviors.^[16,17]^ In contrast, Manlove et al^[18]^ found that shorter sexual relationships were associated with increased condom use and consistency of condom use among male adolescents. This was because many males and females in such relationships do not know their partners’ sexual history.^[18]^ Therefore, associations between types of sexual relationships and subsequent condom use remain unclear. Students’ views regarding responsibility for contraception in different types of sexual relationships have not yet been investigated.

This study aimed to explore male and female students’ individual characteristics, status of condom use, and perceptions of who should be responsible for contraception. Emphasis was placed on the association between students’ perceptions of contraceptive responsibility and condom use in different types of sexual relationships. A deeper understanding of students’ views about contraceptive responsibility may re-frame adolescent pregnancy as an issue for both male and female students, and lead to more effective and gender-inclusive pregnancy prevention and counseling programs.

## 2 Materials and methods

**Figure 1:**
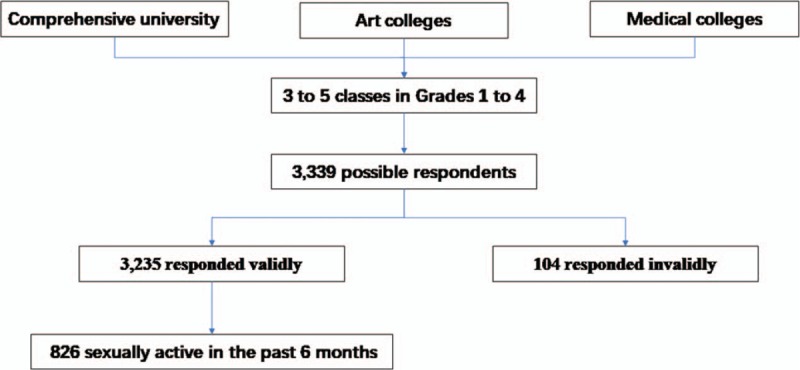
Flow diagram of enrolment of 826 sexually active students during study period.

This study was conducted in Sichuan Province, China, from April to June, 2016. The data were obtained through multistage stratified cluster sampling. Comprehensive university (including majors of science and liberal arts), art, and medical colleges, which were defined by the college entrance examination brochures, were primary sampling units (PSUs). We used simple random sampling to select 1 comprehensive university, 1 school in art, and 1 school in medical. Then, for the second stage, we used simple random sampling to select 3 to 5 classes in each grade in every selected school (approximately 40 students in each class). All students in these classes were recruited. As Fig. 1 shows, of the 3339 respondents who were approached to complete the survey, 3235 responded validly, giving a final valid response rate of 96.9%. Only students who had been sexually active in a heterosexual relationship in the past 6 months were included in the analysis. Of the 3235 respondents, 25.5% (826/3235) met this requirement. In all, 559 of male students and 267 of female students were sexually active in the past 6 months.

For the survey, students were asked to complete the questionnaire by themselves in classroom after a trained staff addressed a brief introduction and instruction. The questionnaire was completely anonymous and should take approximately 10 minutes to complete.

### 2.1 Measurements

The questions were developed based on the related questionnaire which we used in the previous study,^[2]^ and revised by qualitative methods including in-depth interview with 15 students (5 students from each school) and 3 focus group meetings (1 at each school). Then the questionnaire was pilot-tested in a group of 70 randomly selected students in 1 school. The reliability was evaluated by comparing results from 2 surveys with a 2-week interval in 87 college students. More than 70% of questions had Kappa statistics over 0.4 (all *P* < .05). Cronbach alpha coefficient, which was calculated to determine internal consistency of the scales in the questionnaire, ranged from 0.69 to 0.84. Principal components analysis suggested a good fit and the internal design of the questionnaire.

The questionnaire asked students to self-report information including: individual characteristics, their most recent sexual partner (steady and casual), condom use at their most recent sexual intercourse, and contraceptive responsibility for their most recent sexual intercourse. A casual partner (self-identified) was defined as a person involved in a single encounter. A steady partner (self-identified) was defined as a sexual partner who the participant met on a regular basis. The variables measured were contraception, reproductive health consultations, and abortion responsibility based on the following 3 questions:

1. During your most recent sexual intercourse, who ended up being responsible for “taking care” of contraception?
2. During your most recent sexual intercourse, who was responsible for a reproductive health consultation if a contraception problem was encountered?
3. During your most recent sexual intercourse, who was responsible for an abortion (if necessary)?

Response options of the above 3 questions were: “the man,” “the woman,” and “both the man and woman.” Condom use was assessed with the question: “Did you use condoms to prevent pregnancy or STIs during your most recent sexual intercourse?” Response options were “yes” and “no.”

Data were analyzed using Microsoft Excel (2013) and STATA 10.0 (StataCorp LP, College station, TX). Groups were compared with chi-square tests for binary data. Multivariate logistic regression models were used to examine the level of association between individual characteristics, condom use, and students’ views on contraception responsibility. Nominal levels of independent variables with more than 2 categories were transformed into dummy variables and assigned reference categories. We used the adjusted logistic models to calculate multivariable adjusted odds ratios (AORs) for reproductive health-related responsibility by condom use.

The researchers obtained consent from all participants involved in the study. The study was approved by the Research Ethics Committee of West China School of public health, Sichuan University in Chengdu, China. We provided detailed information on the study to the eligible college students and included only those who consented to participate.

## 3 Results

There were 826 complete responses: 559 from men (mean age ± standard deviation [SD] 21.0 ± 1.5 years) and 267 from women (mean age ± SD 20.7 ± 1.5 years) (Table 1). Among them, 60.1% males and 58.1% females reported they had used condoms to prevent pregnancy or STIs at their most recent sexual intercourse. Approximately 80% of sexually experienced students reported their most recent sexual encounter was with a steady partner (79.6% of males and 79.4% of females; Table 1). Over 70% of male and female students believed that the responsibility for reproductive health consulting, contraception, and abortion should be shared.

**Table 1:**
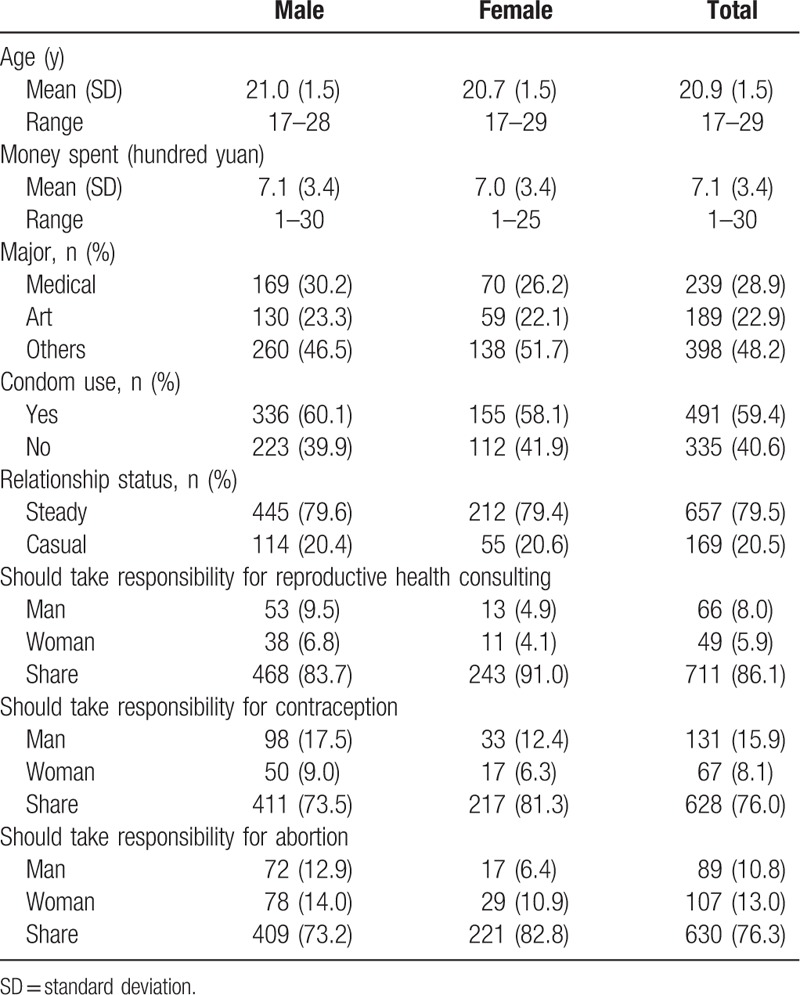
Distribution of dependent variables, individual characteristics, and contraceptive responsibility among male and female college students.

Table 2 shows that male students who were aged ≥20 years were more likely to be in a steady sexual relationship than those younger than 20 years (*P* < .05). Male students with a steady partner had the higher proportion of condom use than those with casual partner. In contact, female students with casual partner had the higher proportion of condom use than those with steady partner. Among students with a steady partner, 90.3% of males and 94.8% of females believed that the responsibility for reproductive health consulting should be shared. Among students with a casual partner, only 57.9% of males and 76.4% of females believed that the responsibility for reproductive health consulting should be shared. Also, 78.4% of male students and 85.8% of female students with a steady partner reported shared contraceptive responsibility. Only 54.4% of male students and 63.6% of female students with a casual partner believed the responsibility for contraception should be shared. Moreover, 78.4% of male students and 85.8% of female students with a steady partner reported shared the responsibility for abortion. Whereas the proportion of male and female students who reported responsibility for abortion should be shared among students with a causal partner were 52.6% and 70.9%. In addition, both male and female students who were in steady relationships had a higher proportion of shared responsibility for reproductive health consultations, contraception, and abortion than those were in casual relationships (*P* < .001).

**Table 2:**
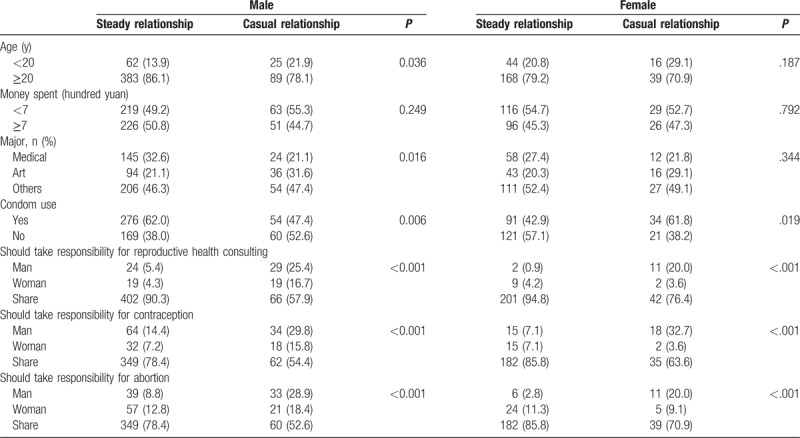
The condom use of students and contraceptive responsibility among students in different types of sex relationship.

Among female students who reported their most recent sexual encounter was with a steady partner, those younger than 20 years were significantly more likely to use condoms than students aged ≥20 years (Table 3). In this group, art school students were more likely to use condoms than students with other majors, and medical students had the lowest rate of condom use. Among male students who were in a casual relationship, condoms were used by 64.5% of those who shared contraceptive responsibility, 26.5% of those who believed contraception was the man's responsibility, and 27.8% of those who believed contraception was the woman's responsibility. In this group, male students who shared responsibility for contraception were more likely to have used condoms at their most recent sexual intercourse than other male students (*P* < .001). Among female students who were in a steady relationship during their most recent sexual encounter, condoms were used by 47.3% of those who shared contraceptive responsibility, 20.0% of those who believed contraception was the woman's responsibility, and 13.3% of those who believed contraception was the man's responsibility. In this group, female students who shared responsibility for contraceptive were more likely to have used condoms at their most recent sexual intercourse than other female students (*P* = .007).

**Table 3:**
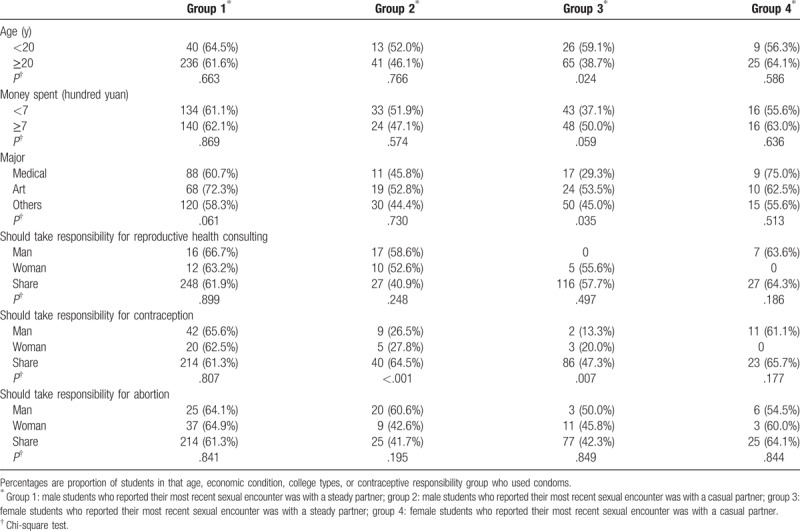
The condom use of students with different types of sex relationship.

Table 4 presents the results from the multivariate logistic regression analyses for the 4 groups. For groups 2 and 3, condom use was associated with greater odds of shared sexual responsibility.

**Table 4:**
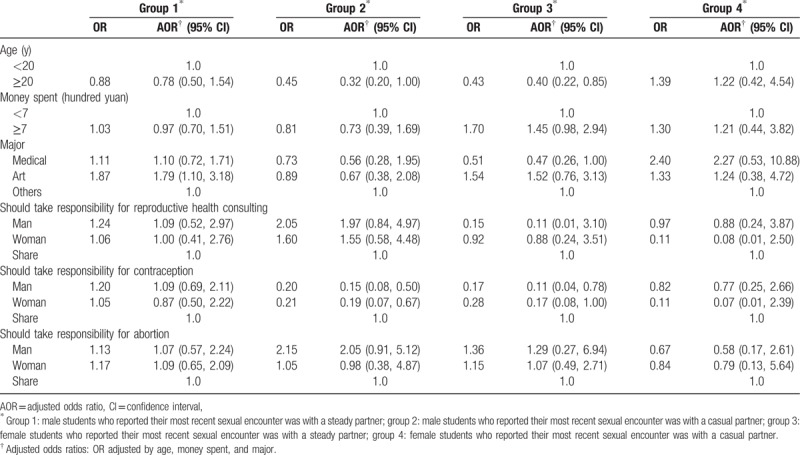
The association between sense of reproductive health-related responsibility and condom use in different types of sex relationship among students.

## 4 Discussion

Despite the growing number and availability of safe and effective contraceptive methods in China, condoms continue to be the most popular form of contraception. In our study, approximately 60% of sexually active students reported that they had used condoms at their most recent sexual intercourse. This rate was higher than that reported in a nationwide survey conducted in China.^[19]^

We found that 17.5% of male and 12.4% of female students believed it was the man's responsibility to “take care” of contraception, and an even smaller proportion believed it was the woman's responsibility (males: 9.0%, females: 6.3%). Although no recent studies of female or male contraceptive responsibility have been conducted in China, this topic has been investigated in other countries. The results of this study supported previous findings in Turkey and the United States that the majority of male and female college students shared contraceptive responsibility.^[13,15]^ In Turkey, 79.4% of male students reported shared responsibility, and 51.8% of female students in the United States reported shared responsibility. In contrast to our results, more students in those 2 previous studies reported contraception was a woman's responsibility. The discrepancy in these findings may reflect differences in culture and prevalence of condom use. Using condoms is a common way for men to share responsibility for contraception in a relationship, and condom use in our study was higher than has been reported in other college populations.^[13]^ This might have influenced women's reports of shared responsibility in their relationships.

Among female students in our study who reported their most recent sexual encounter was with a steady partner, those aged under 20 years were significantly more likely to use condoms than those aged ≥20 years. This might be because younger female students in steady relationships are more likely to use short-term contraceptives or are more concerned about avoiding pregnancy than older female students.^[20,21]^ In addition, among female students who reported their most recent sexual encounter was with a steady partner, art school students were more likely to use condoms than other students, and medical students had the lowest rate of condom use. In contrast, other studies have found that medical students had a higher rate of condom use than other types of students, and art students had a lower rate of condom use than other types of students. This might have been because medical students have more knowledge about contraception, whereas art students are thought to have more open attitudes toward sexual activity.^[4,22]^

We found that condom use was significantly associated with types of sexual relationship. Our study indicated that male students with steady sexual partners were more likely to use condoms than those with casual partners. This finding supported some studies that individuals with causal sexual partners had low rate of condom use, because of the association between reduced communication in early casual relationships and risky sexual behaviors.^[17,23]^ In contrast, we found that female students with casual sexual partners were more likely to use condoms than those with steady partners. This finding supported some studies that casual sexual relationships were associated with increased odds and consistency of condom use because many males and females in this type of relationship do not know their partners’ sexual history, especially those who engage in sexual risk taking.^[18,24]^

In this study, we grouped students by type of sexual relationship to analyze the association between condom use and perspectives on contraceptive responsibility among male and female students. We found that among male students with casual partners and female students with steady partners, condom use was significantly associated with shared contraceptive responsibility. This finding supported the previous research that showed shared responsibility for preventing pregnancy was associated with consistency of condom use.^[20]^ Our study highlighted the need to consider views on shared responsibility for contraception among male students with casual partners and female students with steady partners. Therefore, improving students’ attitudes toward contraceptive responsibility may increase condom use among male students at risk for a partner with an unplanned pregnancy.

There are several limitations to this study. The questionnaire only asked students about condom use. Therefore, we were unable to capture information on use of other contraceptives (eg, oral contraceptives). Furthermore, dimensions of shared contraceptive responsibility are complex and difficult to measure.^[13,25]^ Therefore, we used a qualitative variable to identify if students shared responsibility for contraception. However, shared responsibility may be not equal for men and women, and relationship traits (eg, duration or seriousness of the relationship) may be related to contraception responsibility. Further research is needed to consider these questions. Finally, no cause–effect relationship could be established because of the cross-sectional design of this study. Students provided information on condom use retrospectively. Ideally, condom use would be measured using daily calendars. Despite these limitations, our study extended previous research by identifying a new association between condom use and relationship status among students and how condom usage was related to responsibility.

## 5 Conclusions

Condom use and responsibility for reproductive health, contraception, and abortion have been found closely related to relationship status among male and female college students in our study. The multivariate analysis revealed that condom use is associated with greater odds of shared contraception responsibility in different types of sexual relationships. The findings of our study highlight the need to consider views on shared contraception responsibility in male students with casual partners and female students with steady partners. Improving students’ attitudes toward contraception responsibility may increase condom use among students at risk for unplanned pregnancies. Programs that provide targeted health education and services may help to reduce the high rate of unplanned pregnancies among students in China.

## Acknowledgments

The authors thank all members for their contribution to this research. This study was funded by Fundamental Research Funds for the Central Universities (2017SCU12029).

## Author contributions

**Data curation:** Zhenhua Chen.

**Formal analysis:** Longxia Tong.

**Funding acquisition:** Lu Long.

**Investigation:** Lu Long, Yutong Han, Longxia Tong.

**Methodology:** Lu Long, Zhenhua Chen.

**Project administration:** Lu Long.

**Writing – original draft:** Lu Long, Zhenhua Chen.

**Writing – review & editing:** Lu Long, Yutong Han.
